# Non-invasive evaluation of myocardial reperfusion by transthoracic Doppler echocardiography and single-photon emission computed tomography in patients with anterior acute myocardial infarction

**DOI:** 10.1186/1476-7120-9-16

**Published:** 2011-05-28

**Authors:** Egle Sadauskiene, Diana Zakarkaite, Ligita Ryliskyte, Jelena Celutkiene, Alfredas Rudys, Sigita Aidietiene, Aleksandras Laucevicius

**Affiliations:** 1 Department of Cardiovascular Medicine, Vilnius University Hospital Santariskiu Klinikos, Santariskiu 2, Vilnius, LT-08661, Lithuania; 2 Clinic of Cardiac and Vascular Diseases, Faculty of Medicine, Vilnius University, M.K. Ciurlionio 21, Vilnius, LT-03101, Lithuania

**Keywords:** Myocardial infarction, contractile function, coronary flow reserve, reperfusion

## Abstract

**Background:**

The study was designed to evaluate whether the preserved coronary flow reserve (CFR) 72 hours after reperfused acute myocardial infarction (AMI) is associated with less microvascular dysfunction and is predictive of left ventricular (LV) functional recovery and the final infarct size at follow-up.

**Methods:**

In our study, CFR was assessed by transthoracic Doppler echocardiography (TDE) in 44 patients after the successful percutaneous coronary intervention during the acute AMI phase. CFR was correlated with contractile reserve assessed by low-dose dobutamine echocardiography and with the total perfusion defect measured by single-photon emission computed tomography 72 hours after reperfusion and at 5 months follow-up. The ROC analysis was performed to determine test sensitivity and specificity based on CFR. Categorical data were compared by an χ^2 ^analysis, continuous variables were analysed with the independent Student's t test. In order to analyse correlation between CFR and the parameters of LV function and perfusion, the Pearson correlation analysis was conducted. The linear regression analysis was used to assess the relationship between CFR and myocardial contractility as well as the final infarct size.

**Results:**

We estimated the CFR cut-off value of 1.75 as providing the maximal accuracy to distinguish between patients with preserved and impaired CFR during the acute AMI phase (sensitivity 91.7%, specificity 75%). Wall motion score index was better in the subgroup with preserved CFR as compared to the subgroup with reduced CFR: 1.74 (0.29) vs. 1.89 (0.17) (p < 0.001) during the acute phase and 1.47 (0.30) vs. 1.81 (0.20) (p < 0.001) at follow-up, respectively. LV ejection fraction was 47.78% (8.99) in preserved CFR group vs. 40.79% (7.25) in impaired CFR group (p = 0.007) 72 hours after reperfusion and 49.78% (8.70) vs. 40.36% (7.90) (p = 0.001) after 5 months at follow-up, respectively. The final infarct size was smaller in patients with preserved as compared to patients with reduced CFR: 5.26% (6.14) vs. 23.28% (12.19) (p < 0.001) at follow-up.

**Conclusion:**

The early measurement of CFR by TDE can be of high value for the assessment of successful reperfusion in AMI and can be used to predict LV functional recovery, myocardial viability and the final infarct size.

## Background

Early and successful percutaneous coronary intervention (PCI) is the most effective and preferred reperfusion strategy for treating ST-elevation AMI (STEMI), reducing the infarct size and improving the clinical outcomes. The achievement of an adequate tissue level (myocardial) perfusion is the goal of reperfusion therapy. Nevertheless, myocardial damage is not terminated immediately, even in successful primary PCI with Thrombolysis In Myocardial Infarction (TIMI) flow grade 3 in the infarct-related artery (IRA) [1]. Paradoxically, the restoration of blood flow to the ischaemic myocardium can result in additional cardiac damage and complications, since the introduction of oxygen and energy into an abnormal cellular environment triggers additional events that produce further damage of cells. Manifests the ischaemic-reperfusion (IR) injury which refers to myocardial, vascular and electrical dysfunction [2-6]. Clinical manifestations of the IR injury include:

1. Injury of anatomical and functional integrity of microcirculation, causing the no-reflow phenomenon - inadequate myocardial perfusion of a given coronary segment without angiographic evidence of mechanical vessel obstruction [7-12].

2. Myocardial stunning, which refers to transient dysfunction of myocardium contractility. It results from alterations in contractile proteins due to oxidant stress and/or disturbed cellular calcium homeostasis [13-17].

3. Extension of the infarct zone.

4. Reperfusion arrhythmias (life threatening arrhythmias).

There are several explanations for the impairment of myocardial microcirculation: the plugging of distal segments of the coronary artery tree by microemboli of thrombotic or atherosclerotic debris; endothelial dysfunction which includes increased expression of adhesion molecules, selectins and release of vasoactive substances, resulting in vasoconstriction; increased activation of platelets, neutrophils, the complement system and inflammation; intensified generation of oxygen-free radicals, increased membrane permeability and swelling of endothelial cells and myocites, apoptosis and necrosis of cardiomyocites [18-22]. All these mechanisms can cause microvascular and myocardial dysfunction, deranged myocardial metabolism and more extensive myonecrosis. Previous studies indicate that patients with no-reflow have an increased infarct size, impaired recovery of LV function and poor short- and long-term prognosis [23-29].

The early evaluation of myocardial reperfusion by transthoracic Doppler echocardiography and single-photon emission computed tomography (SPECT) in AMI provides important clues for predicting patient's prognosis [30-32]. The total perfusion defect (TPD) detected by SPECT indicates the extent of no-reflow in the infarct area after reperfusion in AMI and the final infarct size at the follow-up period. The final infarct size is a sensitive end point and can be used to estimate the effectiveness of reperfusion therapy in AMI and to associate with subsequent patient mortality [33-38].

**Figure 1:**
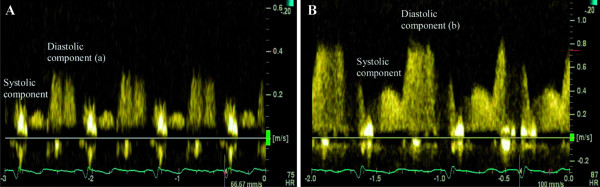
**Coronary flow by spectral Doppler**. The greater component during diastole and the smaller component during systole at rest (A) and during hyperaemia (B). CFR = b/a. CFR > 2 (an example of normal CFR).

The measurement of CFR by TDE has recently entered the stress echo laboratory [39-45]. CFR is expressed as a ratio of hyperaemic to basal peak diastolic coronary flow velocity (Figure 1). The CFR cut-off value of 2 for detecting significant epicardial coronary stenosis and for predicting ischaemia has been demonstrated in various studies. In patients with normal epicardial arteries CFR reflects both structural and functional integrity of the microvasculature in the myocardium. Previous studies revealed that the measurement of CFR in the IRA might reflect a greater degree of microvascular damage in the infarct area, predict myocardial viability and LV remodelling after AMI [46-56]. However, the mean value of CFR varies widely in different studies according to the heterogeneous population studied, the presence and extent of risk factors for vascular dysfunction, the concomitant medical therapy before the test. Moreover, in many cases the efficacy of the reperfusion therapy was assessed quite early after PCI before microvascular damage reached a peak after the reperfusion [57-59]. Therefore, there is no clear cut-off value of CFR during the acute MI phase for the prediction of local and global LV function recovery and the final infarct size.

The aim of our study was to evaluate the association of coronary flow reserve assessed 72 hours after reperfused AMI with myocardial reperfusion and to estimate CFR predictive value for LV functional recovery, myocardial viability and the final infarct size at follow-up.

## Methods

### Study population

The sample of the study was sellected by purposive sampling: all the enrolled patients were treated in the Intensive Care Unit (ICU) of the Department of Cardiovascular Medicine, Vilnius University Hospital Santariskiu Klinikos from February 2008 till September 2010 and fulfilled the inclusion criteria. Totaly 44 patients (6 woman and 38 men, aged 25 - 65 years) were studied. All the patients were with the first anterior STEMI without prior angina pectoris. Inclusion criteria were typical anginal chest pain lasting ≥ 30 min, ST-segment elevation of > 0.2 mV in at least two contiguous electrocardiographic leads, ≥ 3-fold increase in serum creatine kinase (CK) or Troponin I level, angiographically documented single vessel disease with the occluded left anterior descending coronary artery (LAD), with TIMI flow grade 0 - 1 before PCI and TIMI flow grade 3 after PCI. Exclusion criteria were pregnancy, lactation, unstable condition, more than one coronary artery disease, residual LAD stenosis after intervention > 20%, previous myocardial infarction and/or coronary revascularisation, second or third degree atrioventricular block, severe valve disease, systolic blood pressure < 90 mm Hg, bronchial asthma or chronic obstructive pulmonary disease.

### Study design

All the patients underwent revascularization of the LAD by PCI and stent placement using standard technique within 12 hours after the onset of chest pain. Contrast flow through the IRA was graded by the means of the TIMI flow classification. Collateral flow was graded according to the Rentrop classification [60]. 31 patients (70.45%) showed poor or no collateral flow (Rentrop grade 1 or 0) on the initial coronary angiogram. Procedural success was defined as TIMI flow 3 and residual stenosis ≤ 20% in the IRA.

Immediately after PCI, two-dimensional echocardiography (2D Echo) was performed in all the patients in the ICU. LV ejection fraction (LVEF), end-diastolic volume (EDV), end-systolic volume (ESV) were computed by the Simpson biplane method and wall motion score index (WMSI) was calculated using the standard 16-segment LV model [61]. Imaging was performed using the *Vivid S5 System (GE Healthcare, USA).*

The 12-lead electrocardiograms (ECGs) were obtained at the admission, in an hour, 6 hours and 12 hours after the reperfusion. Blood sampling for serum markers of myonecrosis (CK, CK-MB, Troponin I) was routinely taken at the admission, 6 hours, 12 hours and 24 hours after the admission to estimate the peak concentration of these markers.

All the patients underwent 3D Echo, dobutamine stress echocardiography (DSE), CFR, SPECT evaluation 72 hours after the reperfusion. First of all, we performed 3D Echo. Shortly, the myocardial contractility was examined by low-dose dobutamine echocardiography (LDDE). On the same day, several hours after LDDE, adenosine intravenous infusion was administered at a rate 140 μg/kg/min for six minutes for CFR evaluation and for gated-SPECT stress imaging. Spectral Doppler signals of the coronary flow in the distal portion of the LAD were recorded 60 s after the onset of the infusion for CFR off-line analysis. Afterwards, the infusion of adenosine was continued for myocardial perfusion stress imaging. Myocardial perfusion rest images were obtained one day later. The patients were offered chocolate milk to drink before acquisition in order to diminish bowel artefacts. Examinations were performed in the fasting state. Beta-blockers and nitrates were discontinued for 48 hours. No side effects occurred during these procedures. The same examinations were repeated for all the patients at 5 month follow-up.

All the patients gave written informed consent. Study conformed to the principles outlined in the Declaration of Helsinki. Approval of the Lithuanian Bioethics Committee was granted (permission No.2/109, 2008-02-10).

### Electrocardiographic studies

An ECG was recorded using the *Page Writer Trim III *(*Philips Medical Systems 3000, Andover, USA*). The ST-segment elevation (60 ms from the point J) was measured in the single lead (I, aVL, V1-V6) with maximal elevation. The normalization of ST-segment elevation during 1 hour after PCI comparing with the initial ECG was calculated according to the formula:

The ST-segment resolution ≥ 70% was defined as complete resolution, 30-70% - partial resolution and < 30% - no resolution of the ST-segment.

### 3D echocardiography

Real-time 3D Echo harmonic imaging was performed using the *Vivid 7 Dimension Ultrasound System (GE Healthcare, USA) *equipped with a V3 matrix-array transducer. The digital real-time 3D echocardiographic LV data sets were analyzed off-line using *Tom Tec's 4D LV Function *software by semi-automatic detection of LV contours. LVEF, EDV, ESV were computed.

### CFR measurements by transthoracic Doppler echocardiography

The assessment of CFR by TDE was performed using the *Vivid 7 Dimension ultrasound system (GE Healthcare, USA) *with second harmonic technology. For colour Doppler (3 MHz) flow mapping, the velocity range was set at 12 to 20 cm/s. Coronary flow velocity was measured with pulsed wave Doppler at 2.8 MHz (sample volume 2.0 mm) using colour Doppler (CD) as a guide. The angle correction was performed if the angle between the flow and the Doppler beam exceeded 20 degrees and was maintained during rest and stress studies. The spectral trace of the coronary flow was characteristically biphasic with a dominating diastolic component.

The patients were examined in the left position. We recorded the flow in the most distal part of the LAD. A short axis view of the LV and the anterior groove was interrogated with CD. When diastolic CD blood flow was detected, the transducer was slowly rotated clockwise to obtain the best long axis view of the LAD.

**Figure 2:**
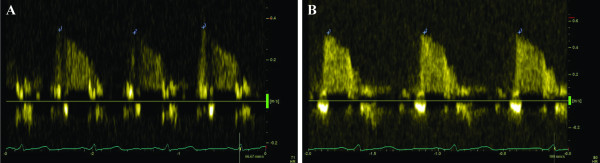
**An example of impaired CFR**. Coronary flow by spectral Doppler at rest (A) and during hyperaemia (B). CFR < 1.5, estimated in the patient with absence of myocardial viability and with extensive myocardial perfusion defect.

First of all, we recorded baseline spectral Doppler signals in the distal portion of the LAD. Then, spectral Doppler signals were recorded during hyperaemic conditions (60 s after the onset of the infusion of adenosine). Stop frames and clips were digitally recorded for off-line analysis. Recordings were done during a brief period of holding breath. The images were interpreted by 2 experienced physicians. Measurements were averaged over three consecutive heart beats. The peak diastolic flow velocity was measured at baseline and at peak hyperaemic conditions. CFR was defined as the ratio of hyperaemic to basal peak diastolic coronary flow velocity (Figure 2).

### Dobutamine stress echocardiography

DSE studies were performed with *Vivid 7 Dimension ultrasound system (GE Healthcare, USA)*. 2D Echo and 12-lead ECG monitoring were performed with low-dose dobutamine infusion. The standard dobutamine stress protocol consisted of continuous intravenous infusion of dobutamine in 3 minutes increments, starting with 5 μg/kg/min and increasing to 10 μg/kg/min. Echo images (apical four- and two-chamber, parasternal long- and short-axis views) were semiquantitatively assessed by 2 experienced physicians using a 16-segment LV model, a wall motion four-grade scale model in resting condition, each stage thereafter and during the recovery phase. We estimated WMSI. In the non-viability response (without contractile reserve), a segment with resting dysfunction remained fixed during stress. If the improvement of wall motion was present in at least four contiguous dysfunctional segments by ≥ 1 grade or WMSI decrease ≥ 0.25 during low-dose dobutamine infusion, they indicated the presence of the stunned myocardium with preserved contractile function which is the predictor of segmental functional recovery at the follow-up period [62]. When DSE was repeated 5 months later, the dose of dobutamine infusion was gradually increased every 3 minutes starting from 5 μg/kg/min to 10, 20, 30 and 40 μg/kg/min. Higher doses were used in order to discriminate between the hibernating (ischaemic) myocardium and myocardial stunning.

### Single-photon emission computed tomography

Gated-SPECT studies were performed using a two-day stress/rest protocol with a dual-detector gamma camera *INFINIA GP 3 (GE Medical Systems, USA)*. ^99 m ^Technetium (Tc) tetrofosmin (750 MBq) was injected intravenously at the third minute of the infusion of adenosine. Supine electrocardiographically gated-SPECT images were acquired after 45 minutes. Rest images were obtained one day later using the same radioactivity of ^99 m ^Tc tetrofosmin with acquisition starting 60 minutes after the injection. The gated and non-gated data were separately reconstructed on a workstation for off-line analysis. No scatter or attenuation correction was applied. The images were interpreted by 2 experienced physicians. We performed both visual semiquantitative analysis, which accounts for a variety of artefactual patterns that can be recognized only by visual inspection, and automatic quantitative computer-generated analysis.

An investigator evaluated visually all images by intensity of myocardial colour-coded scintillation, which depends on the uptake of the radiotracer. The entire LV myocardium was divided into 20 segments. Each segment was scored using a five-point system: the score 0 was used for normal perfusion, i.e. the uptake of the radiotracer was not disturbed; 1 - perfusion was slightly reduced (slight reduction of the uptake); 2 - moderate reduction of the uptake; 3 - severe reduction of the uptake; 4 - no perfusion. Using special software package QPS *(Cedar Sinai Medical Center, Los Angeles, CA, USA) *fully automated quantitative analysis of myocardial perfusion was carried out from "bull's eye" (polar map) images: combined extension and severity of the perfusion defect in one variable - the total perfusion defect in% (TPD%) - was defined at stress and at rest imaging. We referred that TPD < 5% to be normal, between 5 and 9% was termed mild, 10-15% - moderate, and > 15% - severe. We assessed the success of myocardial reperfusion as the TPD (corresponding to final infarct size) by repeated SPECT at follow-up after 5 months.

### Statistical analysis

The statistical analysis was performed with the use of the SPSS software package for Windows 17.0. Data were expressed as mean ± standard deviation (SD) or number (%) of patients. The Receiver Operating Characteristic (ROC) curve analysis was performed to determine test sensitivity and specificity based on CFR. The test performance was estimated by calculating the area under the curve (AUC). The optimal cut-off value of CFR was defined as that providing the maximal accuracy to distinguish between the patients with preserved and impaired CFR during the acute MI phase.

Categorical data were compared by an χ^2 ^(chi square) analysis, continuous variables were analysed with the independent Student's t test. In order to compare WMSI changes separately in the subgroups of patients with preserved and impaired CFR 72 hours after the reperfusion, at LDDE and at follow-up Echo, paired samples t test was conducted. A value of p < 0.05 was considered to be significant.

In order to analyse correlation between CFR and contractile reserve at LDDE, LVEF, TPD at rest and at stress in the infarct area 72 hours after reperfusion and at 5 months follow-up, the Pearson correlation analysis was conducted.

The linear regression analysis was used to assess the relationship between CFR evaluated 72 hours after PCI and preserved myocardial contractility (viability) as well as the final infarct size.

## Results

### The estimation of the optimal CFR cut-off value in order to distinguish between patients with preserved and impaired CFR

**Figure 3:**
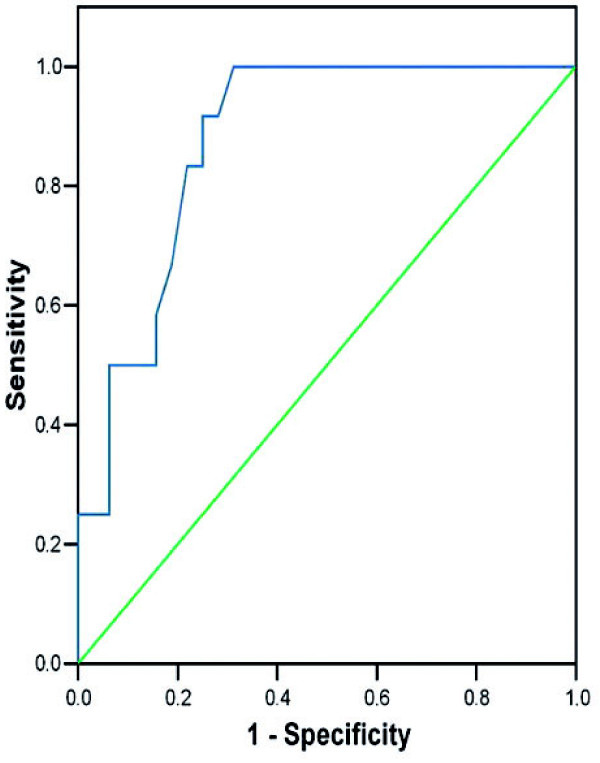
**The ROC analysis for CFR**.

The ROC analysis was performed to estimate test sensitivity and specificity based on a wide range of cut-off points of CFR for the detection of myocardial viability, spontaneous LV functional recovery and a moderate final infarct size. For the evaluation of LV segmental functional recovery, the decrease of WMSI ≥ 0.25 at LDDE during the acute MI phase and at 5 months follow-up echocardiography (as compared to the acute phase echocardiography) was described as a marker of myocardial viability and spontaneous functional recovery. The TPD ≤ 15% was defined as a moderate final infarct size after reperfused MI at follow-up. We estimated the optimal CFR cut-off value of 1.75 as providing the maximal accuracy to distinguish between patients with preserved CFR (N = 19) and impaired CFR (N = 25) during the acute MI phase (sensitivity 91.7%, specificity 75%). The AUC was 0.88 ± 0.1 (p < 0.001) (Figure 3).

### The comparison of the subgroups with preserved and impaired CFR

According to the estimated CFR cut-off value all the patients were divided into two subgroups. Clinical history, risk factors, anti-ischaemic therapy before AMI and angiographic characteristics in both subgroups did not differ significantly, with the exception of timing to reperfusion and ECG ST-segment complete resolution in patients with preserved and impaired CFR (Table 1).

**Table 1:** Clinical and angiographic characteristics in two subgroups of the patients with preserved and impaired CFR

Characteristics		Preserved CFR (N = 19)	Impaired CFR (N = 25)	p value
Age		50.26 (8.46)	51.76 (10.78)	0.620

Gender	Men	16 (84.2%)	22 (88.0%)	0.525

Cigarette smoking		11 (57.9%)	13 (52.0%)	0.467

Hypertension		12 (63.2%)	17 (68.0%)	0.492

Hypercholesterolemia		19 (100%)	25 (100%)	-

Obesity		5 (26.3%)	9 (36.0%)	0.363

ACE inhibitors		6 (31.6%)	5 (20.0%)	0.298

Beta-blockers		4 (21.1%)	8 (32.0%)	0.323

Aspirin		1 (5.3%)	3 (12.0%)	0.415

Diuretics		2 (10.5%)	2 (8.0%)	0.585

Nitrates		0	1 (4.0%)	0.568

Peak CK-MB (U/l)		208.64 (272.20)	428.14 (565.51)	0.127

Peak Troponin I (μg/l)		76.11 (102.66)	130.00 (102.49)	0.084

Time interval from the onset of chest pain to PCI	0-3 hours	12 (63.2%)	7 (28.0%)	
		
	3-6 hours	6 (31.6%)	7 (28.0%)	**0.011**
		
	> 6 hours	1 (5.2%)	11 (44.0%)	

ECG ST-segment complete resolution (≥ 70%) during 1 hour after PCI		9 (47.4%)	4 (16.7%)	**0.033**

TIMI flow grade before PCI	I*	2 (10.5%)	1 (4.0%)	0.396
	
	No flow	17 (89.5%)	24 (96.0%)	

Collaterals		6 (31.6%)	7 (28.0%)	0.528

Data are mean (SD) or number (%) of patients.

ACE - angiotensin converting enzyme; CK - creatine kinase; PCI - percutaneous coronary intervention; ECG - electrocardiography; TIMI - Thrombolysis in Myocardial Infarction; LAD - left anterior descending coronary artery, AMI - acute myocardial infarction.

The majority of patients with preserved CFR (94.8%) received PCI during 6 hours after the onset of chest pain as compared to 56% of patients with impaired CFR. On the contrary, only 5.2% of patients with preserved CFR received PCI after 6 hours from the onset of chest pain as compared to 44% of patients with impaired CFR (p = 0.011). Thus, the timing of reperfusion is very important for the maintenance of the intact microvasculature of the myocardium during AMI. We also revealed statistical difference of ECG ST-segment complete resolution results between the two subgroups: more than 47% of patients with preserved CFR had complete ST-segment regression during 1 hour after PCI as compared to 16.7% of patients with impaired CFR (p = 0.033).

According to these results we can state that an incomplete ST-segment resolution correlates with impaired structural and functional integrity of microcirculation and suboptimal myocardial reperfusion.

### Relationship between CFR, myocardial contractile reserve (viability) and SPECT reflow

**Figure 4:**
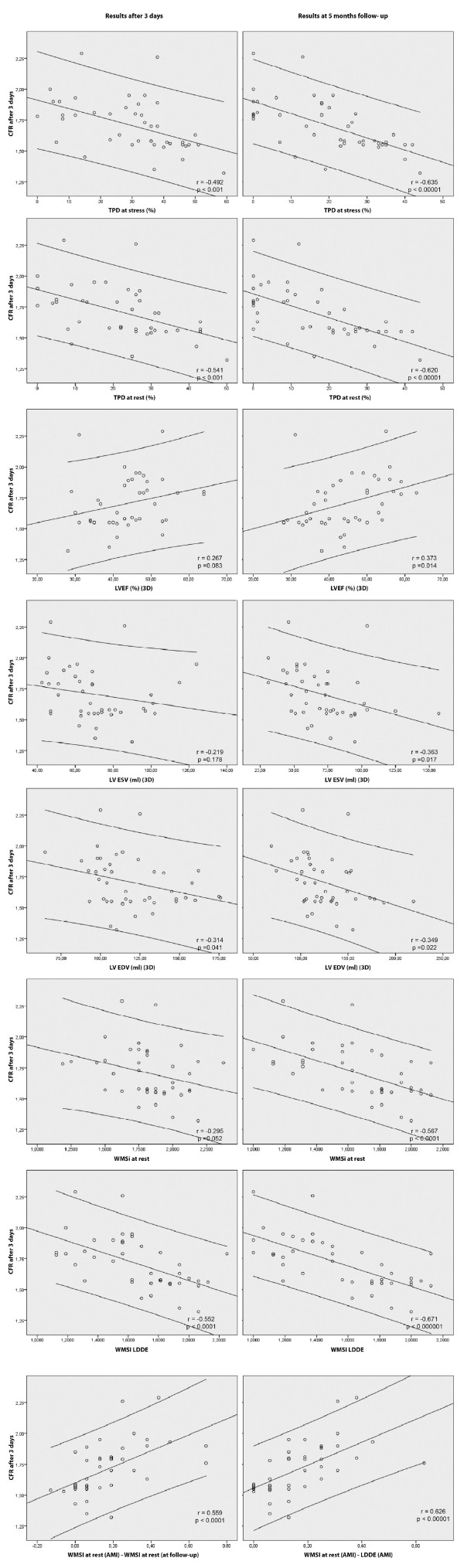
**Correlation between CFR and parameters of LV function and perfusion**. All figures presented in the manuscript are from authors personal archive.

In order to analyse the correlation between CFR assessed 72 hours after reperfusion and the parameters of LV function and perfusion during the acute MI phase and at 5 months follow-up the Pearson correlation coefficient was calculated (Table 2, Figure 4).

**Table 2:** Correlation between CFR and parameters of LV function and perfusion

Parameters of LV function and perfusion	**Correlation coefficient **(**r**)
**72 hours after the reperfusion**	

TPD at stress (%)	-0.492**

TPD at rest (%)	-0.541**

LVEF (%) (3D)	0.267

LV ESV (ml) (3D)	-0.219

LV EDV (ml) (3D)	-0.314*

WMSI at rest	-0.295

WMSI LDDE	-0.552**

**At 5 months follow-up**	

CFR	0.505**

TPD at stress (%)	-0.636**

TPD at rest (%)	-0.620**

LVEF (%) (3D)	0.373*

LV ESV (ml) (3D)	-0.363*

LV EDV (ml) (3D)	-0.349*

WMSI at rest	-0.567**

WMSI LDDE	-0.671**

**Contractile reserve (difference ≥ 0.25 of WMSI)**	

WMSI at rest (AMI) - WMSI at rest (at follow-up)	0.559**

WMSI at rest (AMI) - WMSI LDDE (AMI)	0.626**

WMSI at rest (AMI) - WMSI LDDE (at follow-up)	0.680**

*p < 0.05; **p < 0.001

CFR - coronary flow reserve; LV- left ventricular; LVEF - left ventricular ejection fraction; LV ESV - left ventricular end-systolic volume; LV EDV - left ventricular end-diastolic volume; 3D - three-dimensional echocardiography; WMSI - wall motion score index; WMSI LDDE - wall motion score index at low-dose dobutamine echocardiography; WMSI at rest - WMSI LDDE - difference ≥ 0.25 between WMSI at rest and WMSI at LDDE indicates the presence of contractile reserve; WMSI at rest (AMI) - WMSI at rest (at follow-up) - difference of WMSI ≥ 0.25 indicates the myocardial viability; TPD% - total perfusion defect (%)

A moderate direct correlation between CFR and preserved contractile reserve was observed during the acute MI phase (r = 0.626, p < 0.001) and at follow-up (r = 0.680, p < 0.001), also with spontaneous segmental (r = 0.559, p < 0.001) and global LV (r = 0.373, p < 0.05) functional recovery at the follow-up. CFR was inversely correlated with the TPD which was an indicator of anatomical and functional microvascular integrity and no-reflow extent in the infarct area after reperfusion at rest (r = -0.541, p < 0.001) and at stress imaging (r = -0.492, p < 0.001). Also CFR was inversely correlated with the final infarct size assessed as the TPD at follow-up (r = -0.620, p < 0.001). Most significant correlations were observed between early assessed CFR and the TPD at rest (r = -0.620, p < 0.001) and at stress imaging (r = -0.636, p < 0.001) after 5 months, also with contractile reserve during the acute MI phase (r = 0.626, p < 0.001) and at follow-up (r = 0.680, p < 0.001). There was no statistically significant correlation between CFR and WMSI at rest, LVEF and ESV during the acute MI phase, while correlation between CFR and these parameters was observed after 5 months (r = -0.567, r = 0.373 and r = -0.363, p < 0.05, respectively).

According to these data from our study, CFR measured non-invasively 72 hours after reperfused AMI is an indicator of microvascular integrity and function (the extent of no-reflow), segmental and global LV functional recovery, myocardial viability and the final infarct size.

### The comparison of the parameters of LV function and perfusion in preserved and impaired CFR subgroups

In order to compare the parameters of LV function and perfusion during the acute MI phase and at the follow-up period in preserved and impaired CFR groups, we performed the independent Student's t test analysis (Table 3).

**Table 3:** Comparison of the parameters of LV function and perfusion in two subgroups

Parameters of LV function recovery	Preserved CFR (N = 19)	Impaired CFR (N = 25)	p value
**72 hours after reperfusion**			

TPD at stress (%)	19.68 (12.77)	37.76 (11.59)	< 0.001

TPD at rest (%)	12.58 (1.22)	30.16 (11.32)	< 0.001

LVEF (%) (3D)	47.78 (8.99)	40.79 (7.25)	0.007

LV ESV (ml) (3D)	64.32 (22.97)	74.86 (16.11)	0.084

LV EDV (ml) (3D)	109.06 (21.90)	128.10 (24.15)	0.011

WMSI at rest	1.74 (0.29)	1.89 (0.17)	< 0.001

WMSI LDDE	1.48 (0.27)	1.80 (0.22)	< 0.001

**At 5 months follow-up**			

CFR	2.61 (0.40)	2.06 (0.33)	< 0.001

TPD at stress (%)	8.74 (9.44)	28.12 (9.72)	< 0.001

TPD at rest (%)	5.26 (6.14)	23.28 (12.19)	< 0.001

LVEF (%) (3D)	49.78 (8.70)	40.36 (7.90)	0.001

LV ESV (ml) (3D)	59.28 (2.18)	80.04 (25.10)	0.006

LV EDV (ml) (3D)	113.39 (25.28)	133.72 (3.59)	0.026

WMSI at rest	1.47 (0.30)	1.81 (0.20)	< 0.001

WMSI LDDE	1.30 (0.28)	1.76 (0.25)	< 0.001

**Contractile reserve (difference ≥ 0.25 of WMSI)**			

WMSI at rest (AMI) - WMSI at rest (at follow-up)	0.27 (0.19)	0.07 (0.10)	< 0.001

WMSI at rest (AMI) - WMSI LDDE (AMI)	0.25 (0.13)	0.08 (0.09)	< 0.001

WMSI at rest (AMI) - WMSI LDDE (at follow-up)	0.43 (0.20)	0.13 (0.16)	< 0.001

Data are mean (SD)

CFR - coronary flow reserve; LV- left ventricular; LVEF - left ventricular ejection fraction; LV ESV - left ventricular end-systolic volume; LV EDV - left ventricular end-diastolic volume; 3D - three-dimensional echocardiography; WMSI - wall motion score index; WMSI LDDE - wall motion score index at low-dose dobutamine echocardiography; WMSI at rest - WMSI LDDE - difference ≥ 0.25 between WMSI at rest and WMSI at LDDE indicates the presence of contractile reserve; WMSI at rest (AMI) - WMSI at rest (at follow-up) - difference of WMSI ≥ 0.25 indicates the myocardial viability; TPD% - total perfusion defect (%)

Patients with preserved CFR had a statistically significant smaller TPD than patients with impaired CFR: the TPD was 12.58% (1.22) vs. 30.16% (11.32) (p < 0.001) at rest and 19.68% (12.77) vs. 37.76% (11.59) (p < 0.001) at stress myocardial perfusion imaging during the acute MI phase; the final infarct size was 5.26% (6.14) vs. 23.28% (12.19) (p < 0.001) at rest and 8.74% (9.44) vs. 28.12% (9.72) (p < 0.001) at stress imaging after 5 months, respectively.

Global LV systolic function (LVEF as assessed by 3D Echo) was significantly better in patients with preserved than in patients with impaired CFR: 47.78% (8.99) vs. 40.79% (7.25) (p = 0.007) during the acute MI phase and 49.78% (8.70) vs. 40.36% (7.90) (p = 0.001) at follow-up.

Wall motion and contractility did not improve significantly in patients with impaired CFR with WMSI changing from 1.89 (0.17) 72 hours after PCI to 1.80 (0.21) at LDDE (p = 0.138) and to 1.81 (0.20) at follow-up Echo (p = 0.159). In the group with preserved CFR, WMSI changed significantly from 1.74 (0.29) 72 hours after PCI to 1.48 (0.27) at LDDE (p = 0.009) and to 1.47 (0.30) at the follow-up period (p = 0.008).

Thus, the viable myocardium, spontaneous regional and global LV functional recovery, the smaller no-reflow zone and the final infarct size were more common in patients with preserved CFR than in patients with impaired CFR.

### Relationship between CFR, myocardial viability and the final infarct size

The computed linear univariate regression showed an inverse correlation between CFR assessed 72 hours after AMI and the final infarct size (r = -0.620; R square = 0.385, y = -39.717 x CFR + 83.235), e.g. if a patient's CFR was the same as our study cut-off value of 1.75, the final infarct size was 13.92%. Linear regression also showed statistically significant relation between CFR and preserved contractility (r = -0.680; R square = 0.462, y = 0.751 x CFR - 1.015), e.g. if the patient's CFR was the same as our study cut-off value of 1.75, the difference between WMSI at rest and WMSI at LDDE was 0.2954.

Finally, we analysed the strength of the relationship between early assessed CFR, the final infarct size and myocardial viability (Table 4). The sensitivity varied from 77% to 92% and the specificity - from 66% to 91%. The positive predictive value varied from 50% to 89% and the negative predictive value - from 80% to 95%.

**Table 4:** The relationship between CFR, the final infarct size (TPD) and myocardial viability at follow-up

		TPD yes	≤15% no	Positive predictive value	Negative predictive value	Sensitivity	Specificity
**CFR ≥**	yes	17	2	0,89	0,80	0,77	0,91
					
**1.75**	no	5	20				

		**Myocardial**	**viability**				
						
		yes	no				
					
**CFR ≥**	yes	10	9	0,52	0,92	0,83	0,72
					
**1.75**	no	2	23				

		**Myocardial**	**viability**				
						
		yes	no				
					
**TPD ≤**	yes	11	11	0,50	0,95	0,92	0,66
					
**15%**	no	1	21				

CFR - coronary flow reserve; TPD - total perfusion defect; the difference between WMSI (at rest) during AMI and WMSI (at rest) at follow-up ≥ 0.25 indicates the myocardial viability

According to these data we concluded that preserved CFR during the acute MI phase can predict the preserved myocardial contractility and viability (spontaneous LV functional recovery) and the smaller final infarct size at the follow-up period.

## Discussion

The no-reflow phenomenon was first explored by Ito et al. [25] in patients with anterior AMI after mechanical or pharmacologic revascularization. This phenomenon predicted worse LV functional recovery, increased the risk of the post-infarction complications and poor clinical outcomes [23]. Various subsequent studies [63-67] have proved that preserved microcirculation and reflow after reperfused AMI predict the viable myocardium and the recovery of LV function. Comprehensive and comparative evaluation of different parameters of LV function and perfusion during the acute MI phase and at follow-up was not reported in the literature. Thus, our study is the first complex attempt to assess the influence of impaired microvascular integrity and function as well as the extent of no-reflow on LV contractility, spontaneous functional recovery and the final infarct size by modern, non-invasive techniques.

Previous studies highlighted the importance of non-invasive analysis of CFR in predicting LV functional recovery after coronary reperfusion in AMI [49,51,63,65-67]. However, in all these studies there were no clear cut-off values of CFR, because the mean value of CFR varied widely in different studies due to the heterogeneity of each population studied, different timing of the myocardial reperfusion and the CFR test, residual stenosis after PCI [55,57-59,68]. Therefore, the two subgroups of our study were similar by clinical, medical and angiographic characteristics.

Due to the progression of microvascular damage and reactive hyperaemia after reperfusion in AMI, timing of CFR assessment is very important. Lepper et al. [64] found a significant CFR improvement at 24 hours after reperfused AMI as compared to CFR performed immediately after primary PCI. Rochitte et al. [57] showed that microvascular damage had developed progressively up to 48 hours after reperfusion and stabilized after two days. Accordingly we evaluated myocardial reperfusion by TDE as the CFR measurement and by SPECT as the TPD size 72 hours after successful primary PCI for anterior AMI when microvascular damage after infarction was stable.

The optimal cut-off value of 1.75 to predict myocardial viability, spontaneous LV functional recovery and a moderate final infarct size was proven as that providing the maximal accuracy to distinguish the patients with preserved and impaired CFR. As we predicted, there were differences comparing patients with preserved and impaired CFR according to the time interval from the onset of chest pain to reperfusion. These data was confirmed in other experimental and clinical studies [5,28,29], suggesting that more prolonged and severe ischaemia causes more severe microvascular dysfunction.

Electrocardiographic analysis of ST-segment resolution is one of the simplest and most widely used criterion to assess the effectiveness of restoration of antegrade coronary flow in the IRA and predicts not complicated post-infarction course [69]. However, for a long time there were no clinical studies investigating correlation of ST-segment normalization with the restoration of blood flow in the microvascular bed (tissue reperfusion) and LV functional recovery after reperfused MI. In the multicentre AMICI (Acute Myocardial Infarction Contrast Imaging) trial [70] the investigators found that only TIMI flow grade < 3 and echo contrast defect > 25% were independently associated with LV remodelling at the follow-up period, while ST-segment reduction > 70% was not different between the two groups with and without LV remodelling. These findings were similar in other studies [71,72]. In our study 47.4% of patients with preserved CFR had complete ST-segment regression during 1 hour after PCI as compared respectively to 16.7% patients in the impaired CFR group (p = 0.033). Consequently, the clinical value of ST-segment normalization still remains an object of scientific discussions. According to the data from our study we can conclude that ECG ST-segment normalization is a good indicator of successful tissue reperfusion, but more extensive evaluation is necessary to estimate microvascular integrity and to predict LV functional recovery, the final infarct size and, consequently, a patient's prognosis.

## Study limitations

Our study has been limited by a relatively small number of patients. However, the two study subgroups were quite homogeneous, so the results should be widely applicable. CFR assessment by TDE is a simple, non-invasive technique to obtain the diastolic flow and its velocity in the LAD with the success rate of 90-96%. However, the success rate to obtain the diastolic flow in the right coronary artery is 34-50% and in the circumflex coronary artery - 60-80%, thus restricting its application. Besides, we used dobutamine-induced contractility and wall motion recovery during the acute MI phase and at the follow-up period as indicators of myocardial viability. This approach has been based on semiquantitative analysis, therefore has some limitations when compared with positron emission tomography or magnetic resonance imaging with contrast (quantitative analysis of myocardial perfusion and viability).

## Conclusions

The early, non-invasive measurement of CFR by TDE provides insight into the pathophysiological changes of microcirculatory integrity and function after reperfused AMI. We revealed that CFR measurement can be of high value for the assessment of successful reperfusion in AMI. The preserved CFR with the cut-off value of 1.75 during the acute MI phase can predict the LV functional recovery, myocardial viability and a moderate final infarct size (TPD ≤ 15%) at follow-up with high sensitivity and specificity (91.7% and 75%, respectively). Besides, the combination of the myocardial perfusion test by CFR and SPECT studies as well as the analysis of LV wall motion abnormalities by LDDE represent the evaluation of the flow-function relationship. Concordant contractility and myocardial perfusion results enhance the diagnostic accuracy in the prediction of LV functional recovery after reperfused AMI. Furthermore, the CFR study adds quantitative information to the qualitative assessment of wall motion analysis.

Thus, early assessment of CFR by TDE provides important prognostic information in patients with recent myocardial infarction and should be integrated in the routine evaluation of these patients.

## List of abbreviations

ACE: angiotensin converting enzyme; AMI: acute myocardial infarction; AUC: area under the curve; CD: colour Doppler; CFR: coronary flow reserve; CK: creatine kinase; DSE: dobutamine stress echocardiography; ECG: electrocardiography; EDV: end-diastolic volume; ESV: end-systolic volume; IR: ischaemic-reperfusion injury; IRA: infarct-related artery; LAD: left anterior descending coronary artery; LDDE: low-dose dobutamine echocardiography; LV: left ventricular; LVEF: left ventricular ejection fraction; MBq: megabecquerel; MI: myocardial infarction; PCI: percutaneous coronary intervention; ROC: Receiver Operating Characteristic curve; SD: standard deviation; SPECT: single-photon emission computed tomography; STEMI: ST-elevation acute myocardial infarction; Tc: Technetium; TDE: transthoracic Doppler echocardiography; TIMI: Thrombolysis In Myocardial Infarction; TPD: total perfusion defect; WMSI: wall motion score index; 2D: two-dimensional echocardiography; 3D: three-dimensional echocardiography.

## Competing interests

The authors declare that they have no competing interests.

## Authors' contributions

ES and DZ have been involved in data acquisition, analysis and interpretation, drafted the manuscript. LR and JC participated in drafting the manuscript and revising it. AR and SA were involved in the design of the study. AL conceived of the study, and participated in its design and coordination, and helped to draft the manuscript. All authors read and approved the final manuscript.
